# Complete chloroplast genome of *Exochorda serratifolia* (Rosaceae)

**DOI:** 10.1080/23802359.2022.2098857

**Published:** 2022-07-25

**Authors:** Namsu Jo, Serim Kim, Enkhtsetseg Yeruult, Jin Hee Park, Jungho Lee, Yi Lee

**Affiliations:** a Department of Industrial Plant Science & Technology, Chungbuk National University, Cheongju, Republic of Korea; b Institute for Plant Resources, Memoreal Co., Ltd., Cheongju, Republic of Korea; c Nakdonggang National Institute of Biological Resources, Sangju, South Korea; d Green Plant Institute, Yongin, South Korea

**Keywords:** Chloroplast genome, next-generation sequencing, *Exochorda serratifolia*

## Abstract

*Exochorda serratifolia* (pearlbush) is a rosid shrub found in northeast Asia, including the Korean peninsula. This ornamental plant has white inflorescences and strong insect resistance; however, its genetic diversity is poorly understood and a complete plastid genome is unavailable. Here, we determined the complete chloroplast genome of *E. serratifolia* through *de novo* assembly using next-generation sequencing. The *E. serratifolia* chloroplast genome was 160,558 bp, comprising a large single-copy (LSC) region of 88,514 bp, a small single-copy (SSC) region of 19,308 bp, and two inverted repeat regions (IRs) of 26,368 bp each. We annotated 112 genes: 78 protein-coding genes, 30 tRNA genes, and four rRNA genes. This reference plastid genome increases our understanding of the phylogenetic relationships of *E. serratifolia* among Rosaceae plants.

*Exochorda serratifolia* S. Moore 1877 is a deciduous shrub distributed in northeast Asia, including Liaoning (Qian mountains) in China (Cuizhi and Alexander 2003). It occurs sporadically on the Korean peninsula (Kim et al. 2014). This ornamental shrub has white inflorescences and possesses shade tolerance, freezing tolerance, and insect resistance (Lee et al. 1987). The species is an important genetic resource in Korea, being designated as a biological resource subject to approval for export abroad by the Korean Government. Despite the important economic value of *E. serratifolia* as a genetic resource, studies on its genetic diversity and cultivar identification are very limited. Phenotypic variation among habitats (Song et al. 2016) and ISSR variation among populations (Hong et al. 2013) have been investigated, but not fully resolved. Partial plastid genomic information for this species is available (Zhang et al. 2017); however, characterizing the complete chloroplast genome as a reference is necessary.

Leaf samples were collected from the native habitat in Cheongju, Korea (36° 39′ 03.9″ N, 127° 31′ 16.4″ E), which is designated and protected as a Forest Genetic Resource Reserve (FGRR). A dried voucher specimen was deposited in the Herbarium of the National Institute of Horticulture and Herbal Science, Eumsung, South Korea (IN; http://www.nihhs.go.kr/; voucher number: MPS006522-1; contact: Yoongee Lee, yoong0625@korea.kr). Genomic DNA was extracted using a DNeasy Plant Mini kit (Qiagen, Valencia, CA). A genomic library was constructed using the Illumina HiSeq platform. Raw data obtained through next-generation sequencing were trimmed using the CLC quality trim program (ver. 4.21) in the CLC Assembly Cell package (version 4.2.1, https://www.qiagenbioinformatics.com/products/clc-assembly-cell/), and the chloroplast genome contig was secured and its structure determined through *de novo* assembly. Loci were annotated using the GeSeq program (https://chlorobox.mpimp-golm.mpg.de/geseq-app.html) (Tillich et al. 2017), with manual curation based on BLAST search for final review. The complete chloroplast genome sequence of *E. serratifolia* was deposited in GenBank under accession no. MZ981786.

The complete *E. serratifolia* chloroplast genome was 160,558 bp in length with GC content of 36.5%. The circular structure consisted of a large single-copy (LSC) region of 88,514 bp, a small single-copy (SSC) region of 19,308 bp, and two inverted repeat regions (IRs) of 26,368 bp each. We annotated 112 genes, representing 78 protein-coding genes, 30 tRNA genes, and four rRNA genes.

We performed phylogenetic analysis of 16 species belonging to the Rosaceae, including *E. serratifolia*, using the time-reversal model of the maximum-likelihood algorithm with 1000 bootstrap replications in the QIAGEN CLC Genomics Workbench 21.0 (Hilden, Germany) (https://digitalinsights.qiagen.com/). The phylogenetic tree placed *E. serratifolia* together with the Exochordeae *Prinsepia uniflora* (MZ270554) and close to *Sorbaria sorbifolia* (MN026875) and *Sorbaria arborea* (MN901450) of the Sorbarieae (Figure 1).

**Figure 1.**
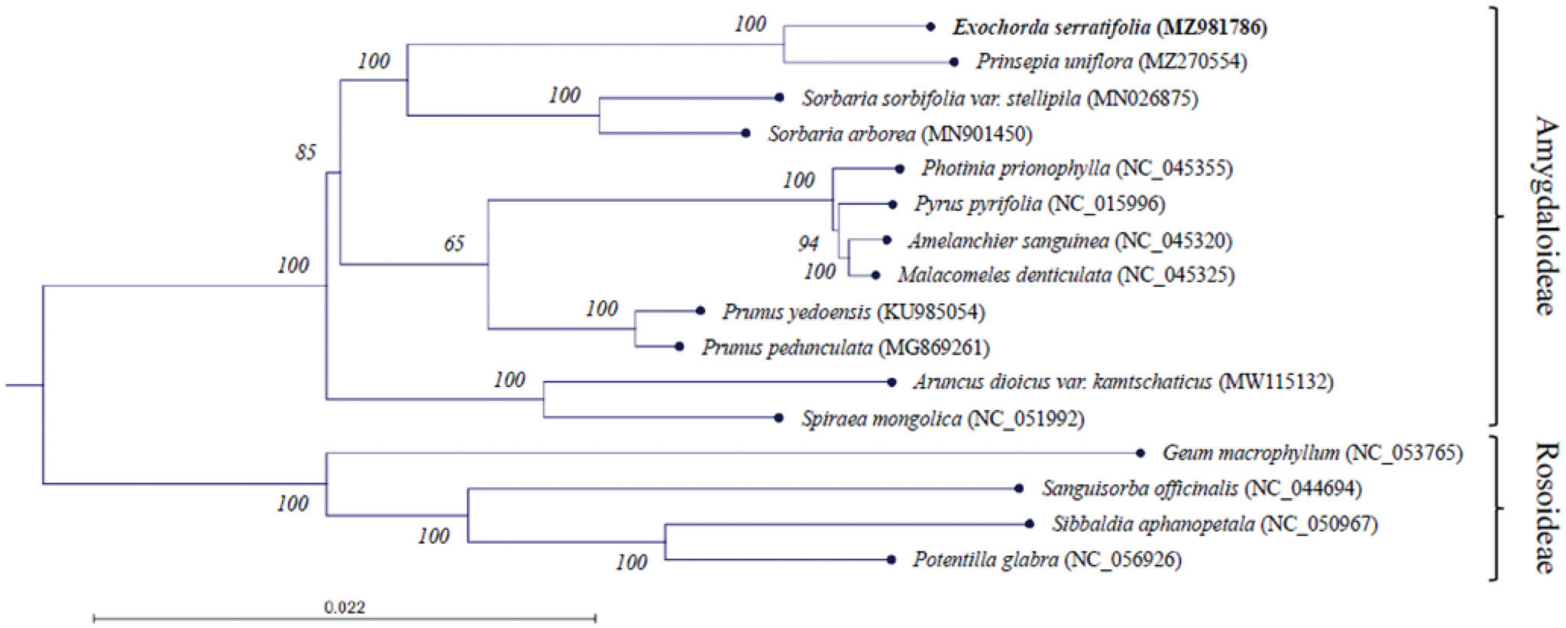
Maximum-likelihood phylogenetic tree based on entire chloroplast genome sequences of *E. serratifolia* and 15 plants belonging to the Rosaceae family. Numbers at nodes represent bootstrap values for 1000 replicates.

## Author contributions

S.K., J.L., and Y.L. conceived and designed the study. S.K., E.Y., J.H.P., and J.L. analyzed the data. N.J. and S.K. performed the experiments. N.J., J.L., and Y.L. wrote the manuscript. All authors read and approved the final manuscript.

## Ethical approval

*Exochorda serratifolia* samples were collected by Serim Kim, a researcher of Memoreal Co., Ltd., with permission from Jinsoon Oh, the Hwajangsa Temple Governor who is responsible for the Forest Genetic Resource Reserve. The plant is freely accessible to Serim Kim with noncommercial resource purpose. The authors comply with relevant institutional, national, and international guidelines and legislation for plant study.

## Data Availability

The genome sequence data that support the findings of this study are openly available in GenBank of NCBI at https://www.ncbi.nlm.nih.gov under the accession no. MZ981786. The associated ‘BioProject’, ‘SRA’, and ‘Bio-Sample’ numbers are PRJN A759290, SRR15681606, and SAMN21162962, respectively.
